# Fishing Hook Globe Injury Diagnosed with Point-of-care Ultrasound: A Case Report

**DOI:** 10.5811/cpcem.53929

**Published:** 2026-07-10

**Authors:** Jeremy Carter, John Robert Zatarain, Mia Zatarain, Paul Koscumb, Krishna Paul, Dietrich Jehle

**Affiliations:** University of Texas Medical Branch, Department of Emergency Medicine, Galveston Texas

**Keywords:** fishing hook injury, traumatic eye injury, point-of-care ultrasound, POCUS, case report

## Abstract

**Introduction:**

Globe injuries constitute true ophthalmologic emergencies and require prompt surgical intervention. When direct physical examination is limited, due to trauma or swelling, point-of-care ultrasound (POCUS) can serve as a valuable adjunct in evaluating globe integrity.

**Case Report:**

We report the case of a 31-year-old male who presented to the emergency department with a barbed fishing hook embedded in his right eyelid. The patient reported pain localized to the eyelid but denied any visual disturbances or direct eye involvement. On examination, a large fishing hook embedded in the right upper eyelid was visualized. His eye was swollen, and he was unable to fully open it, limiting direct assessment of the globe. Given the limited exam, POCUS of the right eye revealed a foreign body traversing the anterior chamber toward the lens, raising concern for globe injury. Ophthalmology was consulted, and computed tomography of the orbits was obtained. The following morning, the patient underwent surgical removal of the barbed fishing hook and repair of the globe.

**Conclusion:**

Point-of-care ultrasonography is a valuable diagnostic adjunct in the evaluation of traumatic eye injuries and useful when physical examination is limited. In this case, POCUS was used to differentiate an intraocular foreign body and globe injury from eyelid foreign body. While POCUS is not routinely recommended in cases of suspected globe rupture, when physical exam is unclear it may be a useful adjunct.

## INTRODUCTION

Globe injuries constitute true ophthalmologic emergencies and require prompt surgical intervention. When direct physical examination is limited due to trauma, swelling, or patient discomfort, point-of-care ultrasound (POCUS) can serve as a valuable adjunct in evaluating intraocular foreign bodies and globe integrity. This is especially important in resource-limited settings where advanced imaging modalities such as computed tomography (CT) or ophthalmology consultation may not be readily available. However, care must be taken to avoid exerting pressure on the globe during ultrasound, as this could worsen the injury. In this case, bedside ultrasound facilitated rapid identification of an intraocular foreign body and probable globe injury, expediting specialty consultation and definitive care.

## CASE REPORT

**Image 1:**
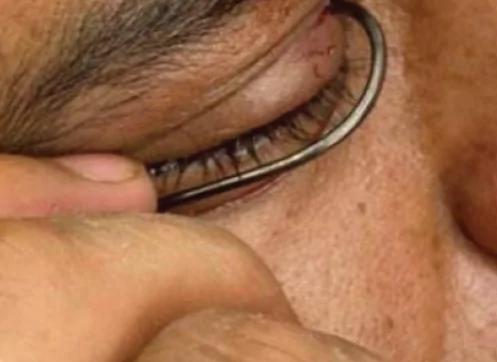
Photo of a fishhook embedded in the right eyelid with concern for globe injury.

A 31-year-old male presented to the ED after sustaining a fishhook injury to his right upper eyelid. The injury occurred while he was fishing. After his hook became lodged in debris, he pulled forcefully on the line, causing the hook to dislodge and embed in his eyelid. The patient reported pain localized to the eyelid but denied any visual disturbances or direct eye involvement. On examination, a large fishhook embedded in the right upper eyelid was visualized (Image 1), Given the limited exam, POCUS of the right eye was performed and revealed a foreign body traversing the anterior chamber toward the lens, raising concern for globe injury (Video).

**Image 2:**
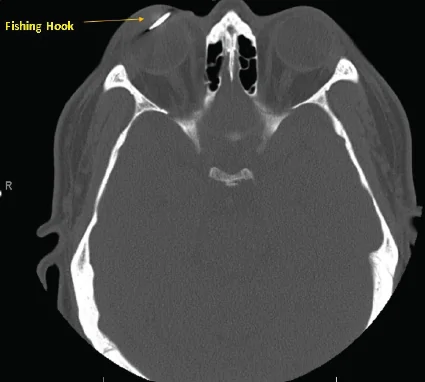
Computed tomography of the orbits, axial image of foreign body in anterior chamber of the right eye.

Ophthalmology was consulted, and a CT of the orbits was obtained to further evaluate the extent of the injury (Image 2). The following morning, the patient underwent surgical removal of the barbed fishing hook and repair of the globe. Three days later, he required a lensectomy due to rupture of the lens capsule.

## DISCUSSION

Open globe injuries with retained intraocular foreign bodies are considered ophthalmologic emergencies. According to the National Library of Medicine, ocular trauma is the leading cause of monocular vision loss in the United States, with open globe injuries being a significant contributor to ocular morbidity and blindness. Recent epidemiological data estimate the incidence of open globe injuries in the U.S. at 4.49 per 100,000 population, with the associated economic burden—primarily from ED visits and hospital admissions—approaching $793 million.^1^ Fishing hooks are a well-documented cause of ocular trauma, particularly in recreational fishing accidents. The mechanism of injury often involves high-velocity retraction of the hook, which can lead to penetration of the eyelid, orbit, or even the globe.^2^–^6^

*CPC-EM Capsule*

What do we already know about this clinical entity?

*Globe Injuries and retained ocular foreign bodies are a surgical emergency. Point-of-care ultrasound (POCUS) can be used as an adjunct to rapidly diagnose the globe injury and foreign body at bedside, when there is clinical ambiguity.*

What makes this presentation of disease reportable?

*In this case, POCUS was used to differentiate an intraocular foreign body and globe injury from eyelid foreign body.*

What is the major learning point?

*While POCUS is not routinely recommended in cases of suspected globe rupture, when physical exam is unclear it may be a useful adjunct.*

How might this improve emergency medicine practice?

*POCUS is routinely used for the emergency diagnoses of multiple patjologies such as retinal detachments,vitreous hemmorhage and lens dislocations. When physical exam is unclear, it can also be used to diagnose foreign bodies and globe injury. Although care must be taken to limit pressure on the eye, which could worsen the injury.*

Point-of-care ultrasonography is a valuable diagnostic adjunct in the evaluation of traumatic eye injuries. It offers high sensitivity and specificity for identifying conditions such as lens dislocation, vitreous hemorrhage, globe rupture, and retrobulbar hematoma. While orbital CT and formal ophthalmologic evaluation remain the gold standard, POCUS is particularly useful when physical examination is limited by swelling, pain, or restricted eyelid movement.^7^–^10^ However, caution must be exercised when using ultrasound in suspected globe injuries. Applying pressure to a compromised globe may lead to extrusion of intraocular contents and worsen the injury. Ultrasound should be avoided in the presence of clear clinical signs of globe rupture, including visible penetrating injury, vision loss, hyphema, or significant periorbital trauma.^11^,^12^

In this case, POCUS was used to differentiate an intraocular foreign body and globe injury from eyelid foreign body. While POCUS is not routinely recommended in cases of suspected globe rupture, when physical exam is unclear it may be a useful adjunct. The patient believed the injury was limited to the eyelid, and the physical exam allowed for the possibility that the globe was uninvolved. The ultrasound exam was performed quickly (< one minute), using a generous amount of gel and applying minimal pressure to avoid further injury. Point-of-care ultrasound aided in prompt diagnosis and escalation of care.

## CONCLUSION

Point-of-care ultrasonography is a valuable diagnostic adjunct in the evaluation of traumatic eye injuries and useful when physical examination is limited. In this case, POCUS was used to differentiate an intraocular foreign body and globe injury from eyelid foreign body. While POCUS is not routinely recommended in cases of suspected globe rupture, when physical exam is unclear it may be a useful adjunct.

## Supplementary Information

Video Point-of-care ultrasound video of the right eye with foreign body traversing the anterior chamber of the eye with concern for globe injury. The blue arrow shows the fishhook entering the anterior chamber and terminating just above the lens. The red arrow shows the posterior chamber.
